# Strain Engineering: A Pathway for Tunable Functionalities of Perovskite Metal Oxide Films

**DOI:** 10.3390/nano12050835

**Published:** 2022-03-01

**Authors:** Samyak Dhole, Aiping Chen, Wanyi Nie, Baeho Park, Quanxi Jia

**Affiliations:** 1 Department of Materials Design and Innovation, University at Buffalo—The State University of New York, Buffalo, NY 14260, USA; sdhole@buffalo.edu; 2 Center for Integrated Nanotechnologies, Los Alamos National Laboratory, Los Alamos, NM 87545, USA; apchen@lanl.gov (A.C.); wanyi@lanl.gov (W.N.); 3 Division of Quantum Phases & Devices, Department of Physics, Konkuk University, Seoul 05029, Korea; baehpark@konkuk.ac.kr

**Keywords:** perovskite, metal oxides, lattice strain

## Abstract

Perovskite offers a framework that boasts various functionalities and physical properties of interest such as ferroelectricity, magnetic orderings, multiferroicity, superconductivity, semiconductor, and optoelectronic properties owing to their rich compositional diversity. These properties are also uniquely tied to their crystal distortion which is directly affected by lattice strain. Therefore, many important properties of perovskite can be further tuned through strain engineering which can be accomplished by chemical doping or simply element substitution, interface engineering in epitaxial thin films, and special architectures such as nanocomposites. In this review, we focus on and highlight the structure–property relationships of perovskite metal oxide films and elucidate the principles to manipulate the functionalities through different modalities of strain engineering approaches.

## 1. Introduction

Perovskite has emerged as an important class of material in technologically important sectors. The research interests and application development of such materials have grown exponentially. Since many elements from the periodic table can be fitted into such a unique crystal structure [1], it is not an overstatement for us to envision that perovskite can be used as a framework to study a wide range of properties and functionalities including ferroic orderings, superconductivity, colossal magnetoresistance, optoelectronics, etc. [2]. Oxide perovskite thin films have been of particular interest in terms of both fundamental research and technological applications for oxide electronics [3]. Since the physical properties of such materials can be further affected by the chemical doping [4,5,6,7] and epitaxial strain [8,9,10,11,12,13], one can strain the materials to tune the properties of the materials towards specific applications. Recently, more approaches for strain engineering have come to the fore. Examples include mechanically straining free-standing single-crystalline membranes [8,14] grown on the buffered substrate where the buffer layer can be etched off thereafter, and the ion implantation of species such as helium [15] and nitrogen [16] to strain the lattice. Perovskite materials have also garnered interest in a wide range of applications such as solar cells, photo/electrocatalysis, photopolymerization, thermoelectrics, resistive switching devices, etc. [17,18,19,20,21,22,23,24,25]. In this review, we will focus on the role of chemical pressure and epitaxial strain in perovskite metal oxide films, superlattices, and vertically aligned nanocomposites.

**Figure 1:**
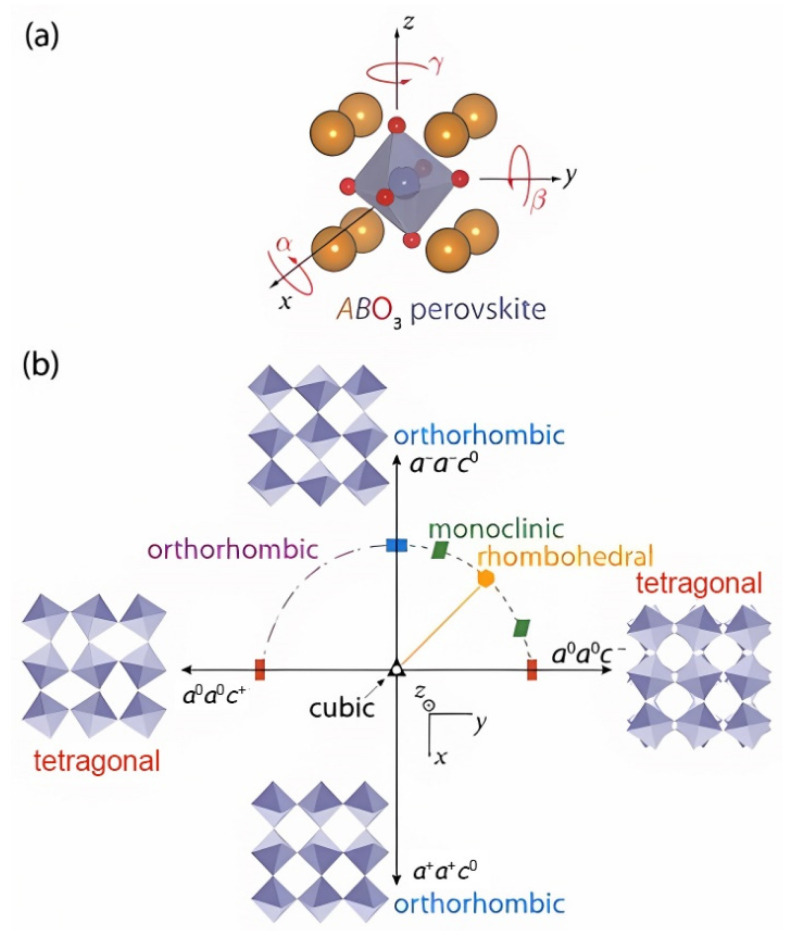
(**a**) The ideal ABO_3_ perovskite crystal structure showing tilt in all three directions. (**b**) Distortion of [BO_6_] octahedra along various directions, lowering the symmetry of the cubic structure and forming other crystal structures. The positive sign indicates in-phase rotation (c^+^) and the negative sign shows out-of-phase rotation (c^−^), both about z-axis. Reprinted with permission from ref. [26]. Copyright 2011, Wiley.

The ideal structure of perovskite with a chemical formula ABO_3_ is cubic, as shown in Figure 1a, and composed of a three-dimensional network of corner-sharing [BO_6_] octahedra, where the A cation sits in the cubo-octahedral site between adjacent octahedra. However, the structures of most perovskite materials exhibit a lower symmetry, such as rhombohedral, orthorhombic, or tetragonal [2]. The geometry and symmetry of the crystal structure are directly affected by the relative size of the comprising ions and often viewed through the proxy of Goldschmidt tolerance factor, t:

(1) $$t=\frac{R_{A}+R_{X}}{\sqrt{2\left(R_{B}+R_{X}\right)}}$$

For the ideal cubic aristotype, t=1. As t lowers further, the structures distort via octahedral tilts and rotations, as shown in Figure 1b, to lower the symmetry until the perovskite phase stops being stable [2,26]. From the viewpoint of chemical compositions, the structure can therefore be distorted by doping isovalent elements of different sizes to exert a chemical pressure on the lattice.

Another way to affect the crystal structure is through epitaxial strain. While bulk crystals are brittle and fragile to mechanical strain, epitaxial thin films can tolerate strains of the order of several percent [27,28]. Such epitaxial thin films have a rich history in perovskite metal oxides facilitated by the advances in thin-film growth techniques such as molecular beam epitaxy, pulse laser deposition, sputtering, metal–organic chemical vapor deposition, and chemical solution deposition. Biaxial strains in perovskite metal oxide films can be used as a lever to tune physical properties such as electric polarization, magnetoresistance and magnetic anisotropy, and stabilize metastable phases [9]. Strain can distort the perovskite structure by tilting, rotating, and elongating the oxygen octahedra, and therefore significantly affect the properties of given materials such as Curie temperature (T_C_) [10,29,30,31], magnetic anisotropy [32,33,34,35,36,37], and magnetotransport properties of ferromagnetic materials [38,39,40,41]. In ferroelectric perovskite oxides, for example, the epitaxial strain can enhance the T_C_ for several hundreds of degrees [11,42,43,44,45]. One of the best illustrations is probably SrTiO_3_, where the lattice strain can make SrTiO_3_ ferroelectric at room temperature, despite the unstrained SrTiO_3_ being paraelectric throughout all temperatures [13]. Similarly, epitaxial strain-engineering can also be leveraged in multilayer epitaxial heterostructures, i.e., epitaxial layered structures, superlattices, and vertically aligned nanocomposites (VANs). For example, ferroelectric SrTiO_3_ has been experimentally demonstrated in fully strained SrTiO_3_ films [13], superlattices SrTiO_3_/BaZrO_3_ [45] and SrTiO_3_/BaTiO_3_ [11], and vertically aligned epitaxial SrTiO_3_:MgO and SrTiO_3_:Sm_2_O_3_ nanocomposite films [46,47].

## 2. Strain Engineering through Chemical Substitution or Chemical Pressure

The substitution of ions via chemical pressure is one of the common approaches to strain the crystal structure. As the A-site cations prefer to form close-shelled electronic configurations with fixed valency, they largely play a structural role. The cation size influences lattice constants, bond lengths and angles, and octahedral rotations and tilts. In other words, a distortion of the central [BO_6_] octahedra can directly affect the material properties. Both the mean size and the size mismatch of the cations on the A-site affect the electronic and structural phase transitions [48,49,50]. 

**Figure 2:**
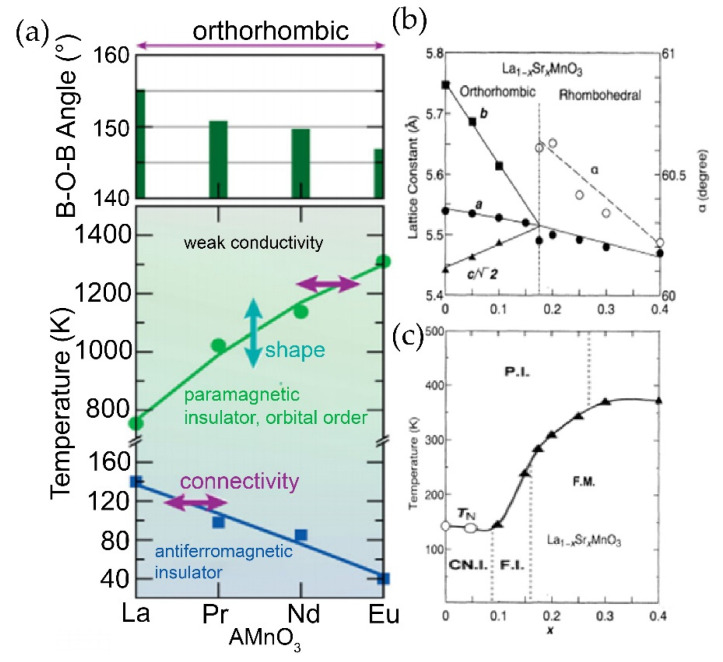
(**a**) The isovalent phase diagrams of rare-earth manganites with A-site composition exhibit changes in the octahedral connectivity (B-O-B bond angles, upper panels) and symmetry. Changes to the octahedral connectivity (horizontal arrows) affect the temperatures associated with magnetic and charge-ordering phase transitions, whereas periodic orderings of the octahedral shape and size (vertical arrows) occur at the orbital-ordering transition. Reprinted with permission from ref. [51]. Copyright © 2022, The Materials Research Society. (**b**) Lattice parameters for La_1−x_Sr_x_MnO_3_ crystals at room temperature. (**c**) Electronic phase diagram of La_1−x_Sr_x_MnO_3_. Open circles and filled triangles are the Neel (T_N_) and Curie (T_C_) temperatures, respectively. The abbreviations mean paramagnetic insulator (P.I.), paramagnetic metal (P.M.), spin-canted insulator (CN.I.), ferromagnetic insulator (FI), and ferromagnetic metal (FM). Reprinted with permission from ref. [52]. Copyright 1995, American Institute of Physics.

To illustrate strain engineering to tune the functionalities of perovskites through chemical substitution or chemical pressure, we use AMnO_3_ manganates as the model system. Mixed-valent perovskite manganates, AMnO_3_, are one of the most widely studied systems for their colossal magnetoresistance (CMR) property. The Curie temperature of these materials, T_C_, is very sensitive to the change in chemical pressure which can be introduced via the substitution of trivalent rare earth metals of different sizes. For example, as the atomic radius of the A cation changes from a relatively larger to a smaller size in the case of La, Pr, Nd and Eu, and the Neel temperature, T_N_, for the antiferromagnetic insulator phase is lowered with the change in the B-O-B angle away from 180°, as shown in Figure 2a [51]. In Ln_1−x_M_x_MnO_3_ systems, where Ln = trivalent lanthanide (La, Pr, Nd, …), M = divalent cation (Ca, Sr, Ba, …), the disorder due to size disparity between the two A-site cations plays a role in determining and tuning their electronic properties. This is evident from the fact that different Ln_1−x_M_x_MnO_3_ perovskites with the same doping level and tolerance factor can have quite different metal-to-insulator transition temperatures [48]. As the x value increases, the hole doping increases linearly. The average A cation size also changes linearly with x value, but the change in disorder is non-linear, with it being minimum at x = 0 and 1, and maximum at x = 0.5. The statistical variance between the distribution of the radii can be considered as the quantity to parametrize this disorder [48]. La_1−x_Sr_x_MnO_3_ is the most well studied material in this class. Here, the bond lengths and angles are lowered with an increase in Sr content. The effect of Sr content on lattice parameters and bond angle is depicted in Figure 2b [52]. The change here is even more dramatic compared to the prior example of switching trivalent rare-earth metals. As shown in Figure 2c [52], the T_N_ first begins to decrease until a transition to a ferromagnetic insulator phase, and eventually to a ferromagnetic metal with T_C_ of the ferromagnetic phase well above room temperature. The pressure exerted in the lattice modifies local parameters such as the Mn-O bond length and the O-Mn-O bond angle which, in turn, affects the balance between the co-existing metallic and insulating states, and thus their CMR properties [53]. Mixed-valent perovskite manganates also exhibit many charge-ordered phases that are affected by factors such as the size of the A cations as well as isotopic and chemical substitutions [54,55,56].

Another well studied system is the titanate, ATiO_3_ (A = Ba, Sr, Ca), where the ferroelectric Curie temperature, T_C,_ was seen to be manipulatable by the size variance of the A-site cation mixture [57]. Ba_1−x_Sr_x_TiO_3_ shows a decrease in T_C_ with an increase in Sr^2+^ as the smaller cation stabilizes the more symmetric cubic phase with tolerance factor decreasing from that of the ferroelectric BaTiO_3_, which has a tolerance factor of 1.06. However, in Ba_1−x_Ca_x_TiO_3_, the size mismatch between the A cations is bigger and only x up to 0.24 is stable without phase segregation. The T_C_ rises to x = 0.08 from 403 K to 410 K, and then decreases to the solution limit. This behavior cannot be explained by simple size arguments, where the initial increase has been rationalized by the strain effect due to the mismatch between the two cations and the subsequent decrease due to the size effect [49,57].

One of the major limitations of chemical substitution to exert chemical pressure over epitaxial strain engineering is the introduction of disorder and heterogeneity associated with chemical substitution in such films. Disorder, for example, can broaden the phase transition by hundreds of degrees [58,59,60]. We will discuss the epitaxial control of strain in the following sections.

## 3. Epitaxial Strain Engineering

**Figure 3:**
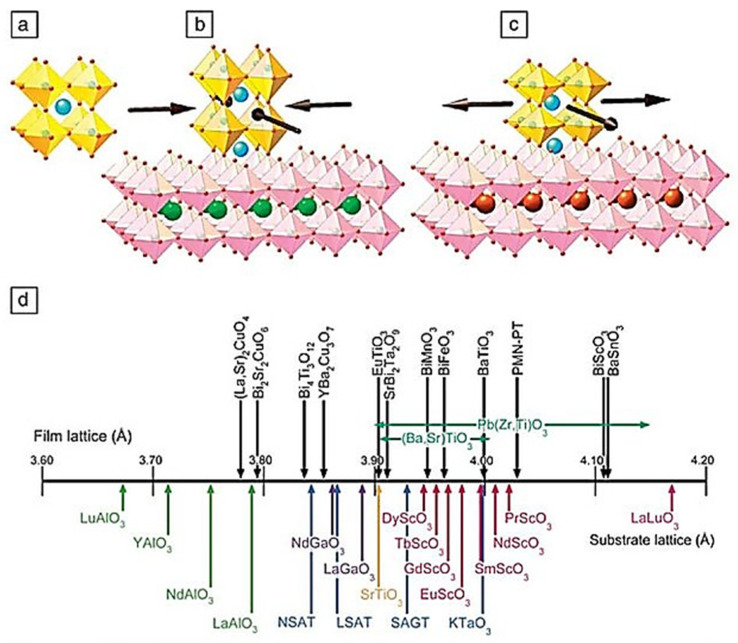
(**a**) Crystal structure of an unstrained perovskite. (**b**,**c**) Schematic illustration of an epitaxial perovskite film grown on a perovskite single-crystal substrate showing: (**b**) biaxiall compression, (**c**) biaxial tension, (**d**) the a-axis lattice constant in angstroms of some perovskites and perovskite-related materials of interest. The substrates can be cubic, pseudotetragonal or pseudocubic. Reprinted with permission from ref. [9]. Copyright 2014, Cambridge University Press.

A range of commercially available single-crystal substrates with different lattice parameters makes it possible to epitaxially grow perovskite oxide thin films with different strain states. The lattice mismatch and the strain between the substrate and the film can then be designed by selecting an appropriate substrate. Other considerations besides the lattice parameters are chemical and thermal expansion compatibility. The most commonly used and commercially available substrates and thin-film materials of interest are shown in the lower part of Figure 3d. Lots of materials of interest (top part of Figure 3d) can be epitaxially grown on these substrates with certain lattice mismatches.

For a simple cubic lattice, the lattice mismatch, f, between the substrate and the film can be defined as:

(2) $$f=\frac{a_{s}-a_{0}}{a_{0}}$$

where $a_{s}$ and $a_{0}$ are the lattice parameters of the substrate and film in an unstrained state, respectively. In a fully strained epitaxial film, the in-plane lattice parameters of the film are constrained to the lattice of the substrate. However, as the thickness of the film increases above a critical film thickness, defects such as misfit dislocations become energetically favorable, and the lattice strain relaxes. 

For isotropic films, the biaxial strain is:

(3) $$\varepsilon_{xx}=\varepsilon_{yy}=\frac{a_{∥}-a_{0}}{a_{0}}$$

and the out-of-plane strain is:

(4) $$\varepsilon_{zz}=\frac{a_{⊥}-a_{0}}{a_{0}}$$

where $a_{∥}$ and $a_{⊥}$ are the in-plane and out-of-plane lattice parameters of the strained epitaxial film. A substantial portion of the change in lattice parameters due to epitaxial strain is through changes in the relative magnitude of the [BO_6_] octahedral rotations and the B–O bond lengths [51,61].

The strain on the film evolves with the increase in thickness of the film, as the top of the film starts to relax with dislocation defects and the strained in-plane lattice parameter starts to converge to the relaxed bulk value, deviating from that of the film–substrate interface, eventually reaching full relaxation [62]. The strain in the film leads to the storage of elastic energy and at the critical thickness, this energy equals the formation energy for misfit dislocation defects in the People–Bean model [62]. Critical thickness, therefore, is inversely correlated with the lattice mismatch [63]. However, the critical thickness can also differ depending on experimental growth conditions such as growth temperature, post growth annealing, stoichiometry, etc., due to the influence of thermal strains and defect formation. Thermal strains arise due to a mismatch between the in-plane thermal expansion coefficients between the two lattices during the cool-down process [64,65,66]. In cases of post-growth annealing, which are often used to decrease the number of oxygen vacancies and improve the crystallinity of the film, the detailed annealing protocol could also change the strain state in the as-deposited films. For instance, in the deposition of epitaxial films of La_0.7_Sr_0.3_MnO_3_ on Al_2_O_3_/MgO substrate–buffer platform, it has been reported that the post-growth annealing temperature beyond the deposition temperature of ~900 °C could create an irreversible strain relaxation and degrade the magnetic saturation of the films. The properties of the films were only partially recovered by a second annealing step at 700 °C [67].

**Figure 4:**
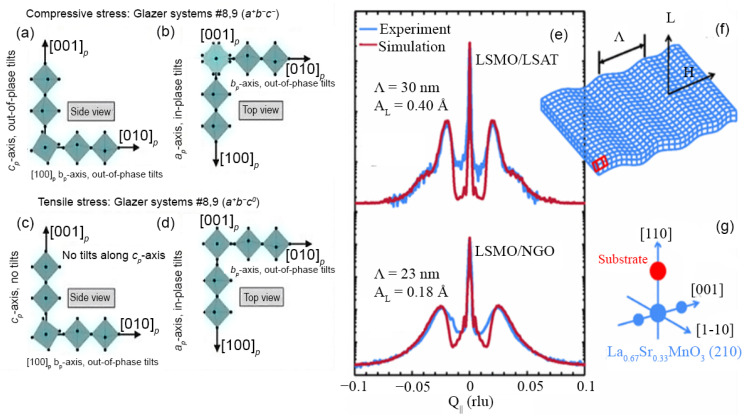
Schematic representation of octahedral rotational patterns in La_0.67_Sr_0.33_MnO_3_. (**a**,**b**) Under compressive stress. (**c**,**d**) Under tensile stress. (**a**,**c**) are the side view and (**b**,**d**) are the top view of the unit cells. Reprinted with permission from ref. [68]. Copyright 2015, Elsevier Ltd. Lattice modulations: (**e**) Observed (blue) and simulated (red) XRD profiles around LSMO (220) reflections for films grown on (La, Sr)(Al, Ta)O_3_ (LSAT) and NdGaO_3_ (NGO) substrates. (**f**) Schematic drawing of the lattice modulations used in calculations. (**g**) Schematic picture of a reciprocal space showing the substrate peak (red) together with the LSMO (220) peak and its first-order satellites (blue). Here, Q_∥_ = 4πsin(θ/λ), where θ is the Bragg angle and λ = 1.540598 Å. Reprinted with permission from ref. [38]. Copyright 2011, American Physical Society.

Vailionis et al. studied the strain accommodation in the rhombohedral La_0.67_Sr_0.33_MnO_3_ (LSMO) via lattice modulations and rotations [38,68]. Under compressive strain, LSMO has an (110) out-of-plane-oriented monoclinic unit cell with space group P21/m (No. 11), while under tensile strain it exhibits an (001) out-of-plane-oriented tetragonal unit cell with space group Cmcm (No. 63). Under compressive strain, out-of-phase octahedral rotation around the (001) direction occurs, while under tensile strain these rotations are absent. The octahedra are rotated in phase around the (100)-axis of the pseudocubic unit cell and out of phase around the (010) direction in both cases (Figure 4a–d). The additional strain along the (100)-direction is accommodated by periodic lattice modulations, as shown in Figure 4f. The changes in octahedral rotations owing to stress and the dissimilar in-plane rotational patterns affect the in-plane magnetic anisotropy in LSMO films. Similar results were seen for the orthorhombic SrRuO_3_, and the authors argued that this could be extrapolated to other rhombohedral and orthorhombic perovskite oxides [38]. Strain accommodation via similar lattice modulations has also been experimentally observed by high-resolution transmission electron microscopy in epitaxial YBa_2_Cu_3_O_7−δ_ with the presence of twin boundaries and intergrowths [69,70]. High-resolution transmission electron microscopy has emerged as a powerful tool towards enabling the direct observation of such structural changes [71]. In ultrathin La_2/3_Sr_1/3_MnO_3_ films grown on NdGaO_3_ substrates, strong oxygen octahedral coupling is found to transfer the octahedral rotation in the perovskite substrate to the perovskite thin film near the interface [72]. An unexpected realignment of the magnetic easy axis along the short axis of the unit cell as well as the presence of a giant anisotropic transport in these ultrathin La_2/3_Sr_1/3_MnO_3_/ NdGaO_3_ films was observed. Similar control over octahedral tilts was also demonstrated in SrRuO_3_ by using a 0–4 unit cell thick Ca_0.5_Sr_0.5_TiO_3_ buffer layer on GdScO_3_ substrates [73]. The Ru–O–Ti and the Ru–O–Ru bond angles at the interface could be tuned via changing the thickness of the buffer layer, along with affecting magnetic anisotropy in the entire SrRuO_3_ layer.

**Figure 5:**
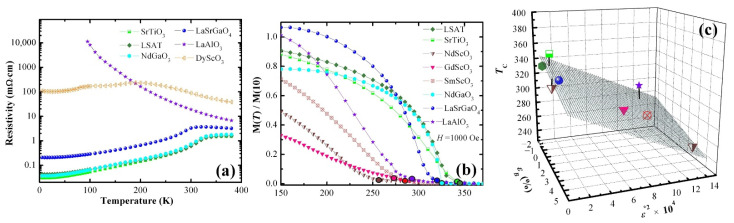
(**a**) Resistivity vs. temperature behavior, at zero applied magnetic field, of 22 nm thick La_0.7_Sr_0.3_MnO_3_ films on different substrates (10 nm thick on NdScO_3_). (**b**) The temperature dependence of the magnetization normalized at 10 K of samples cooled in a 1000 Oe field of *H* = 1000 Oe. Circles indicate the Curie temperature, T_C_. (**c**) The Curie temperature T_C_ vs. the ε_B_ and ε* strains. The best fit plane to the data is also shown. Reprinted with permission from ref. [30]. Copyright 2009, American Institute of Physics.

In (001) LSMO, the resistivity of thin films has been shown to be controlled via epitaxial strain. The effect on the temperature dependence of resistivity can be seen in Figure 5a [30]. Metal-to-insulator transition can be lowered considerably by using substrates that can lead to a higher tensile strain. This can be clearly seen from a systematic comparison of the resistivity vs. temperature characteristics of films on different substrates such as SrTiO_3_ (0.5%), DyScO_3_ (1.6%) GdScO_3_ (2.3%), SmScO_3_ (2.7%) and NdScO_3_ (3.2%). The Curie temperature and magnetization were also seen to be dependent on substrate choice (Figure 5b). In accordance with theoretical predictions by Mills et al. [74], a best-fit plane of the Curie temperature’s dependence on the bulk compressive strain $\varepsilon_{B}\;$(which tends to increase electron hopping probability and reduce the effects of electron–phonon coupling) and biaxial distortion ε* (which increases the Jahn–Teller splitting in the e_g_ orbitals and acts only to reduce T_C_) is shown in Figure 5c.

Interestingly, in lightly doped manganites, the in-plane compressive strain has been found to increase the T_C_ significantly and produce metal-insulator-transition (MIT) in the compounds that are supposed to be ferromagnetic insulators (FMI) [60]. For example, Bulk La_0.9_Sr_0.1_MnO_3_ is a ferromagnetic insulator with a Curie temperature (T_C_) of 145 K. When grown epitaxially on LAO, thinner La_0.9_Sr_0.1_MnO_3_ (~15 nm) films are metallic with a greatly enhanced T_C_, which is 97 K higher than the bulk value. In-plane compressive strain (−1.5%) was reported to be partially responsible for the T_C_ enhancement. Strain-induced stoichiometry modification also plays a role in modulating the T_C_ and strain relaxation in these thin films, accommodated both by misfit dislocations and La deficiency. 

The opposite effect of compressive in-plane strain on optimally doped and lightly doped manganites is not surprising. In optimally doped manganites, the in-plane compressive strain induces the change in the O–Mn–O bond angle and length away from the ideal case. This, therefore, reduces T_C_ and MIT. It is noted that this strain in lightly doped manganites induces oxygen octahedral tilt and straightens the out-of-plane O–Mn–O chain which promotes MIT. 

**Figure 6:**
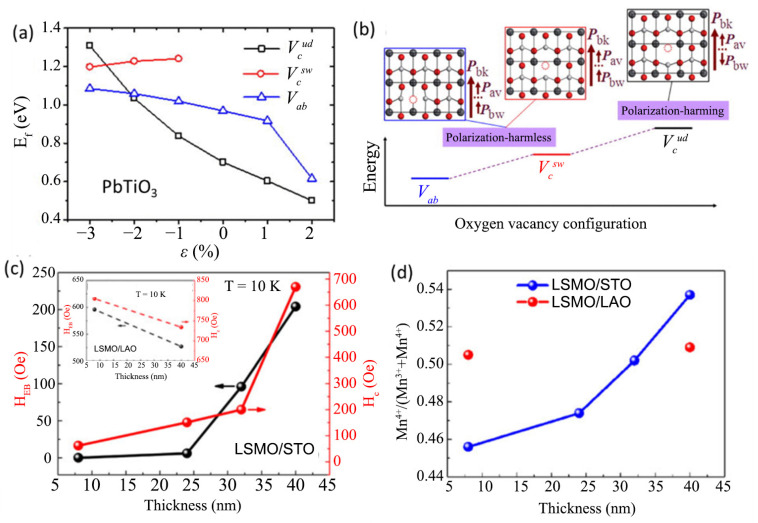
(**a**) Formation energies (E_f_) of different types of oxygen vacancy under different strains. The black, red, and blue symbols represent E_f_ of $V_{c}^{ud}$, $V_{c}^{sw}$, and $V_{ab}^{\;}$, respectively. The formation of the “up-down” pattern along the x-axis causes the sudden decrease in energy of V_ab_ when 2% tensile strain is applied. (**b**) The relative energy stabilities and the polarization patterns of different oxygen vacancy configurations in 3% compressive-strained PbTiO_3_. Reprinted with permission from ref. [65]. Copyright 2013, American Institute of Physics. (**c**) Thickness dependence of H_C_ (right axis) and H_EB_ (left axis). For comparison, inset shows H_C_ and H_EB_ of La_0.5_Sr_0.5_MnO_3_ films grown on the LaAlO_3_ substrate with the thickness of 8 and 40 nm (**d**) The thickness dependence of Mn^4+^/(Mn^3+^ + Mn^4+^) ratio in La_0.5_Sr_0.5_MnO_3_ films grown on the SrTiO_3_ substrate. The red dots correspond to the data of La_0.5_Sr_0.5_MnO_3_ films grown on the LaAlO_3_ substrate for comparison. Reprinted with permission from ref. [67]. Copyright 2016, Materials Research Society.

Lattice strain has also been implicated in oxygen vacancy formation which can lead to radical changes in the physical properties of these films. For instance, oxygen vacancies may act as pinning sites for ferroelectric and magnetic domain wall movements, create structural disorder and introduce electronic defects [75,76,77]. In the prototypical ferroelectric perovskite system, PbTiO_3_, ab initio study showed that oxygen vacancies that act as strong domain pinning centers can be modulated to a non-pinning center via compressive misfit strain by changing the formation energies of the various types of oxygen vacancies, as shown in Figure 6a,b [78]. Ferroelectric distortion stabilized in SrTiO_3_ by epitaxial strain has also been shown to promote the formation of oxygen vacancies [79]. In half-doped manganite, La_0.5_Sr_0.5_MnO_3_, the epitaxial strain was shown to modulate ferromagnetic and antiferromagnetic phase proportions by manipulating the oxygen nonstochiometry as evidenced by the change in Mn^3+^ and Mn^4+^ ratio with the increase in thickness (Figure 6d). This was seen to lead to depressed magnetization and enhanced exchange bias (Figure 6c) [80]. In La_0.67_Ca_0.33_MnO_3_, the epitaxial strain was implicated in the formation of a dead layer near the interface with a higher concentration of oxygen vacancies with reduced Mn valence and the unidirectional displacement of Mn ions [81].

**Figure 7:**
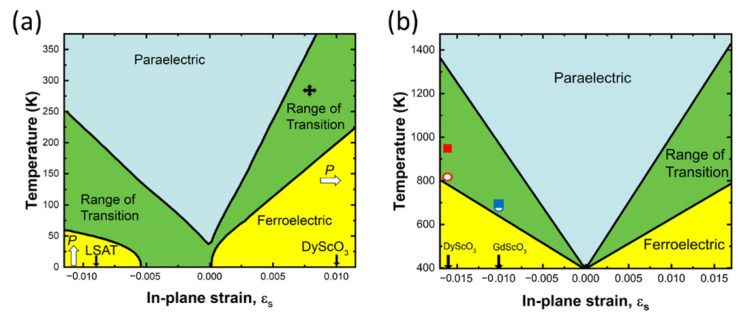
(**a**) Expected shift in T_C_ of (100) SrTiO_3_ with biaxial in-plane strain, based on thermodynamic analysis. The arrows indicate the predicted direction of the polarization for strained SrTiO_3_: in-plane for biaxial tensile strain and out-of-plane for biaxial compressive strain. The ε_s_ values for SrTiO_3_ fully constrained (commensurate) to the lattice constants of LSAT and (110) DyScO_3_ substrates are indicated by the positions of the corresponding arrows. The cross shows the observed T_C_ shift of a 500 Å thick SrTiO_3_ film epitaxially grown on (110) DyScO_3_. Adapted with permission from ref. [13]. Copyright 2004, Springer Nature. (**b**) Expected T_C_ of (001) BaTiO_3_ under biaxial in-plane strain (ε_s_), based on thermodynamic analysis. The green region represents the range (error bars) in the predicted T_C_ resulting from the spread in reported property coefficients for BaTiO_3_ that enter into the thermodynamic analysis. The data points show the observed ε_s_ and T_C_ values of coherent BaTiO_3_ films grown by MBE on GdScO_3_ (blue circle) and DyScO_3_ (red circle) substrates and by PLD on GdScO_3_ (blue square) and DyScO_3_ (red square) substrates. Adapted with permission from ref. [11]. Copyright 2004, AAAS.

The tuning of T_C_ in ferroelectric systems via epitaxial strain can be well illustrated in the titanates. The much enhanced ferroelectric property is most explicitly demonstrated by the case of SrTiO_3_, which is normally not ferroelectric at any temperature without any lattice strain. However, when SrTiO_3_ is grown epitaxially on DyScO_3_, with an in-plane tensile strain of ~0.94%, fully strained SrTiO_3_ film shows a T_C_ as high as 293 K [13]. This strained SrTiO_3_ film was later demonstrated to be an orthorhombic phase [82], and showed an antiferrodistortive phase transition that exhibited ferroelastic–ferroelectric multiferroicity [83]. The strain vs. Curie temperature phase diagram of SrTiO_3_ predicted from thermodynamic principles using a Landau–Ginsburg–Devonshire-type theory by Persev et al. is shown in Figure 7a. It illustrates the complex interconnection between the Curie temperature and the lattice strain [13,84]. 

**Figure 8:**
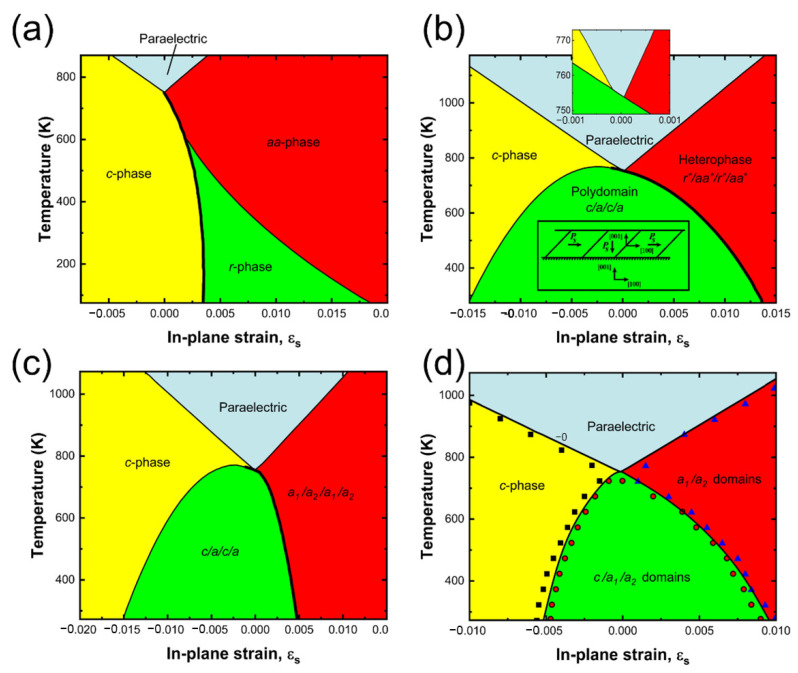
Four strain-phase diagrams of (001) pseudocubic-oriented PbTiO_3_ calculated using thermodynamic analysis or phase-field simulations and different assumptions of the ferroelectric domain states. (**a**) Single domain for all ferroelectric states. Adapted with permission from ref. [92]. Copyright 1998, American Physical Society. (**b**) Either single- or double-domain states with domain–wall orientations restricted to be 45° from the film/substrate interface. Adapted with permission from ref. [88]. Copyright 2000, American Physical Society. (**c**) Single- or double-domain states with domain–wall orientations restricted to be either 45° or 90° from the film/substrate interface. Adapted with permission from ref. [89]. Copyright 2001, American Physical Society. First-order phase transitions are shown by thick lines in (**a**–**c**). (**d**) From three-dimensional phase-field simulations that automatically predict the possible multidomain states without assuming the domain–wall orientations. Adapted with permission from ref. [93]. Copyright 2008, American Institute of Physics.

The coupling between the lattice strain and the ferroelectric properties such as Curie temperature is common in perovskite metal oxides. For instance, the Curie temperature and the ferroelectric polarization of the classical ferroelectric perovskites BaTiO_3_ and PbTiO_3_ can also be influenced by lattice strain. BaTiO_3_ thin films coherently grown on GdScO_3_ and DyScO_3_ substrates with a misfit strain of about −1.0% and −1.7%, respectively, show a large increase in ferroelectric transition temperature with a T_C_ 400 °C on GdScO_3_ and 540 °C on DyScO_3_. In comparison, the T_C_ is only 120 °C for bulk BaTiO_3_. It was also observed that the strained thin film had a remnant polarization 250% higher than bulk BaTiO_3_ single crystals (Figure 7b) [11]. These results are comparable to unstrained Pb(Zr_x_Ti_1−x_)O_3_, but with a lead-free composition, which is preferable due to environmental and human health implications of the processing and disposal of the toxic element. Theoretical studies also suggest that there are temperature and strain regions, in particular, under tensile strain, where the system decomposes into multi-domain structures [85,86,87]. Despite both BaTiO_3_ and PbTiO_3_ being titanates, the primary drivers of ferroelectric polarization differ. For BaTiO_3_, the polarization arises from the off-centering of the Ti^+4^ cation, while the 6s^2^ lone pair on Pb^2+^ in PbTiO_3_ is stereoactive and contributes to the ferroelectric polarization too. Different approximations and assumptions about domain configurations in thermodynamic analysis and phase-field simulations of strain-phase diagrams of PbTiO_3_ lead to markedly different phase diagrams, as shown in Figure 8. All the diagrams involve the paraelectric phase, the tetragonal c-phase, and are under the assumption of a single-domain film. The orthorhombic aa-phase exists at higher in-plane strains with a ‘‘monoclinic gap’’ r-phase between the tetragonal and orthorhombic phases (Figure 8a). However, real films always have more than one type of domain and extended phase diagrams that account for two-dimensional domains are subsequently derived from thermodynamic calculations of Pertsev et al. (Figure 8b,c) [88,89]. Subsequently, phase-field models, which could predict multi-domain states without making assumptions about domain wall orientation, were used to derive the three-dimensional phase diagram shown in Figure 8d. Experimental and theoretical calculations concur that the T_C_ can be increased via both tensile and compressive strains. It is noted that the T_C_ is also evidently affected by the film thickness. The increase in T_C_ with film thickness indicates that the ferroelectric property is further affected by grain size in addition to the lattice strain [90,91].

## 4. Superlattices

**Figure 9:**
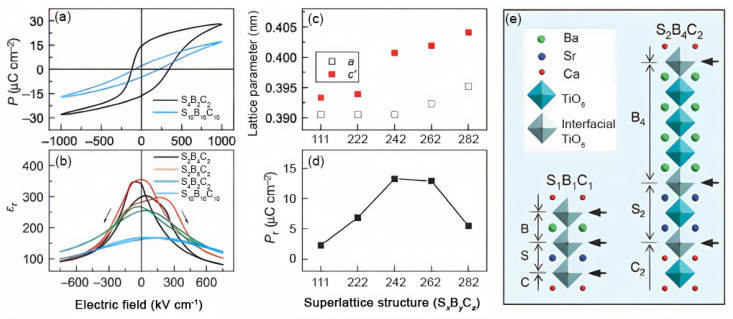
(**a**) *P*(*E*) curves of S_2_B_4_C_2_ (*P*_r_ ≈ 16.5 µC cm^−2^) and S_10_B_10_C_10_ (*P*_r_ ≈ 3.5 µC cm^−2^) at 1000 kV cm^−1^. (Note that this electric field is higher than that applied to measure the polarization of the BaTiO_3_ film, whose polarization is near saturation at 400 kV cm^−1^, whereas the S_2_B_4_C_2_ requires a higher electric field (∼700 kV cm^−1^)). (**b**), ε(*E*) curves of S_2_B_4_C_2_ (black line), S_2_B_6_C_2_ (red line), S_4_B_2_C_2_ (green line) and S_10_B_10_C_10_ (light blue line) showing differing degrees of asymmetry. (**c**) In-plane (open squares) and out-of-plane (filled squares) lattice parameters of various superlattices. *c*′ corresponds to the supercell *c* divided by the number of constituent perovskites in a supercell. (**d**), *P*_r_ values from *E* = ±750 kV cm^−1^ loops. The partially relaxed S_2_B_8_C_2_ structure was measured with *E* = ±650 kV cm^−1^ because of its lower breakdown strength. (**e**) Diagrams of supercells showing the different local environments possible for the TiO_6_-octahedra (bound by the same or different A-site cations). Heterointerfacial TiO_6_ octahedra are shaded in grey and indicated by solid black arrows. Reprinted with permission from ref. [94]. Copyright 2010, Springer Nature.

In layered structures and superlattices, strain relaxation behaviors are different from single-layer systems. The lattice mismatch between the layers and their thicknesses can all contribute to the strain state of the whole structure. For instance, strain engineering of superlattices such as BaTiO_3_/SrRuO_3_ can be achieved by choosing suitable substrates and controlling the thickness of each component layer [63]. One of the remarkable examples of superlattices and their unique interplay with strain is seen in the system of (SrTiO_3_)/(BaTiO_3_)/(CaTiO_3_), where the BaTiO_3_ layer shows strain-induced ferroelectric polarization with 50% enhancement of the superlattice global polarization compared to a pure BaTiO_3_ layer grown similarly (Figure 9) [94]. The BaTiO_3_ layer remains fully strained as long as the number of unit cells does not exceed that of the combined SrTiO_3_ and CaTiO_3_. Figure 9c shows how the lattice parameter of the superlattice shifts as a function of the stacking sequence. Their polarization is depicted in Figure 9d. Polarization is strongest when the BaTiO_3_ layer is thin enough to be fully strained but thick enough to contain enough non-interfacial TiO_6_ octahedra. The advantage of such superlattices is the possibility of breaking inversion symmetry that persists with most two-component or symmetric superlattices [95], and provides additional freedom in tuning the average lattice parameter [94]. The interrogation of ε(*E*) curves shown in Figure 9b illustrates asymmetric response due to compositionally broken symmetry.

## 5. Vertically Aligned Nanocomposites

As discussed above, epitaxial strain provides an alternative pathway to chemical doping for manipulating the physical properties of a range of perovskite metal oxide films. Experimental results have indeed demonstrated that much improved physical properties and/or emergent behaviors could be accomplished by growing lattice-strained epitaxial perovskite metal oxide thin films [11,13,94,96,97,98]. However, enhanced functionalities are typically achieved in such epitaxial metal oxide films with a thickness of less than a few tens of nanometers at which the lattice strain could be maintained [11,13,97,98,99].

For many technological applications, lattice-strained thick epitaxial perovskite metal oxide films (in the range of ~µm or above) are needed. In a conventional lattice strain framework, heteroepitaxial strain resulting from the lattice mismatch between the film and the substrate exists only below a critical film thickness. In other words, films are strained to the substrate lattice up to a critical thickness, h_c_. Above h_c,_ the strain energy becomes so large that the nucleation of misfit dislocations at the interface is then energetically favorable to relieve the strain. The critical thickness, h_c_, at which the formation of dislocations becomes favorable, can be expressed as:

(5) $$h_{c}=\frac{b}{4\pi f\left(1+v\right)}\left[\ln(\frac{h_{c}}{b})+1\right]$$

where v is the Poisson ratio, b is the magnitude of the Burgers vector, and f is the lattice mismatch between the film and the substrate. As can be seen from Equation (5), reducing the lattice mismatch could circumvent the upper limit of critical film thickness to some extent.

**Figure 10:**
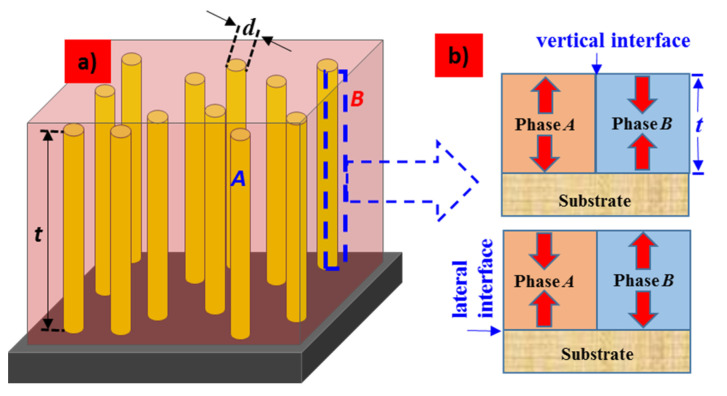
Schematic illustration (not to scale) of a vertically aligned epitaxial nanocomposite film on a substrate: (**a**) an array of vertically aligned nanopillar-like material *A* with a feature size *d* entrenched in a matrix of material *B* and (**b**) the lattice–strain interactions across the vertical interface. For simplicity, both phases are shown to be strained equally, but in different strain states (compression or tension).

Great success in the lattice strain of much thicker films has been demonstrated in the vertically aligned nanocomposite, where the lattice strain is predominantly controlled by the vertical interface between the two individual constituents instead of the lateral interface between the film and the substrate. As shown in Figure 10a, vertically aligned nanocomposite film is composed of a regularly arranged *A:B* network from an array of vertically aligned nanopillar-like material *A* with a feature size *d* entrenched in a matrix of material *B*. Since both constituents *A* and *B* are laterally coupled across the vertical interface through the whole film thickness, as shown in Figure 10b, the vertical strain can exist in thick films. For example, lead-free, self-assembled BaTiO_3_:Sm_2_O_3_ nanocomposite films (up to 1.25 µm thick) were grown that exhibited tetragonality up to at least 800 °C and strong remanent polarization to at least 330 °C [100]. Similar nanocomposites, such as Ba_0,6_Sr_0.4_TiO_3_:Sm_2_O_3_, with film thickness in the range of 1.0 µm were also demonstrated that showed tetragonality up to at least 400 °C [101].

**Figure 11:**
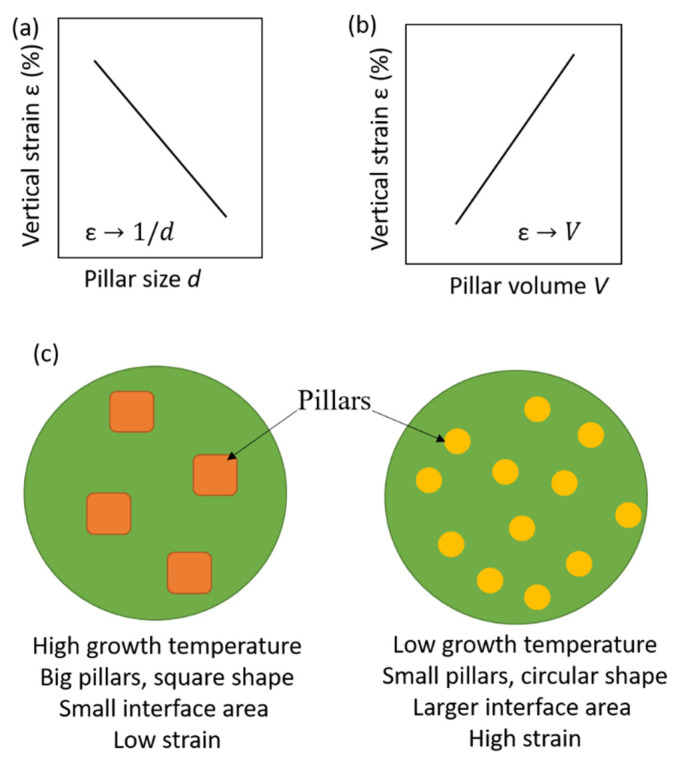
(**a**) The pillar-size-dependent vertical strain in the nanocomposite film with a fixed pillar volume. In this case, ε is proportional to 1/*d*. (**b**) Pillar-volume-dependent vertical strain in the nanocomposite film with a fixed pillar size. In this case, ε is proportional to the pillar volume *V* when *V* is below 20–30 percent. (**c**) Illustrations of growth temperature, pillar size and strain state in the nanocomposites. Reprinted with permission from ref. [102]. Copyright 2021, Springer Nature.

Characteristics of lateral epitaxial strain are well studied. However, the relationship between vertical strain and microstructure in vertically aligned nanocomposites is less known. It is important to know the parameters that control the vertical strain and microstructure of the nanocomposites. The final microstructure (pillar size, shape, and lateral spacing) of a vertically aligned nanocomposite film is determined by the minimization of the total free energy, which includes the elastic and interfacial energies of the system composed of the two individual phases (*A* and *B*) and the substrate. At the given growth conditions, both the size and volume of pillars predominantly determine the interfacial area at the vertical interface. It is accepted that the pillar size *d*, the density of the nanopillars, as well as the defects along the vertical interface determine the overall strain state of vertically aligned nanocomposites [32]. Figure 11 shows the general relationship between the vertical strain, the pillar feature size, and the volume of pillars [102]. Figure 11 also shows the general correlation among the growth temperature, pillar size, and strain state. In a system with a fixed second-phase volume, a higher growth temperature will generally produce a larger pillar size. This will result in a smaller interface area, which usually leads to a lower vertical strain. On the other hand, a lower growth temperature generally results in a smaller pillar size and larger interface area, which can lead to a larger vertical strain. In a system with a fixed pillar size, the increase in the pillar density can increase the total vertical interface area and thus lead to a larger strain. It was reported that the vertical strain in vertically aligned nanocomposites is ultimately related to the vertical interfacial area and interfacial dislocation density [12,32]. This can be understood by the following. In a nanocomposite with uniform nanopillars embedded in the film matrix, the total volume of nanopillar phase is *V* = *n × πd*^2^/4 × *h*, where *n* is the number of nanopillars, and *h* is the film thickness or pillar height. The total vertical surface area can be expressed as *S* = *n* × *πd* × *h* = *πd* × 4*V*/(*πd*^2^) = 4*V*/*d.* Since Figure 11 shows strain ε is proportional to 1/*d* and *V* then, ultimately, strain is directly controlled by the vertical interface area. The efficiency of the vertical interface coupling is related to the coherence of the interface.

Table 1 and Table 2 have summarized some vertically aligned nanocomposite films with the inclusion of perovskite metal oxides reported in the literature [32]. For most material systems, the assembly occurs as a result of nucleation and growth, with embedded pillars of the elastically stiffer phase in the matrix of the softer phase or the perovskite oxides. Pseudospinodal decomposition is the other growth mode which can lead to a checkerboard configuration which is associated with symmetry-lifting crystal lattice rearrangement [103,104].

**Figure 12:**
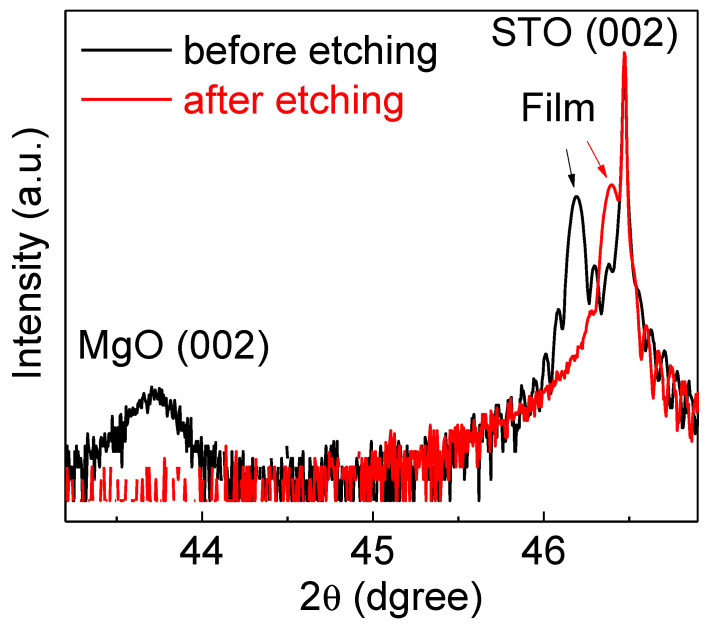
X-ray diffraction θ–2θ scans of SrTiO_3_:MgO vertically aligned nanocomposite films deposited on SrTiO_3_ substrate before and after etching off MgO nanopillars. Reprinted with permission from ref. [11]. Copyright 2019, Wiley.

Such vertical interface strain has been used to tune functional properties in oxides. Room-temperature ferroelectricity in vertically strained SrTiO_3_ nanocomposite with MgO nanopillars embedded in the strained SrTiO_3_ matrix has been demonstrated [46]. An out-of-plane strain of ~1.5% was observed in the film matrix. Elastic coupling between MgO and SrTiO_3_ can be confirmed by etching away the MgO pillars, after which the lattice parameter of the SrTiO_3_ relaxes to 3.911 Å, as shown in Figure 12 [12]. The measured strain was corroborated by phase-field modeling to explore the spatial distribution of the strain and the resultant polarization. The T_C_ was extracted from optical second-harmonic generation (SHG) measurements. The phase transition was found to be very broad, which was consistent with the non-uniform strain distribution and the non-uniformity shown in the piezoelectric force microscopy measurement.

A multiferroic epitaxial nanocomposite of CoFe_2_O_4_ nanopillars in a matrix of BaTiO_3_ was demonstrated [118]. The CoFe_2_O_4_ nanopillars experienced an out-of-plane compressive strain of 0.8%. The coupling between the ferroelectric perovskite BaTiO_3_ and the ferrimagnetic spinel CoFe_2_O_4_ allowed the interconversion of energies stored in electric and magnetic fields to create an emergent ferroelectromagnet. The magnetoelectric coupling could be explained by the strong elastic interactions between the two phases by thermodynamic analyses, which was also further supported by the fact that the coupling could not be reproduced in multilayer structures due to the substrate clamping effect.

**Figure 13:**
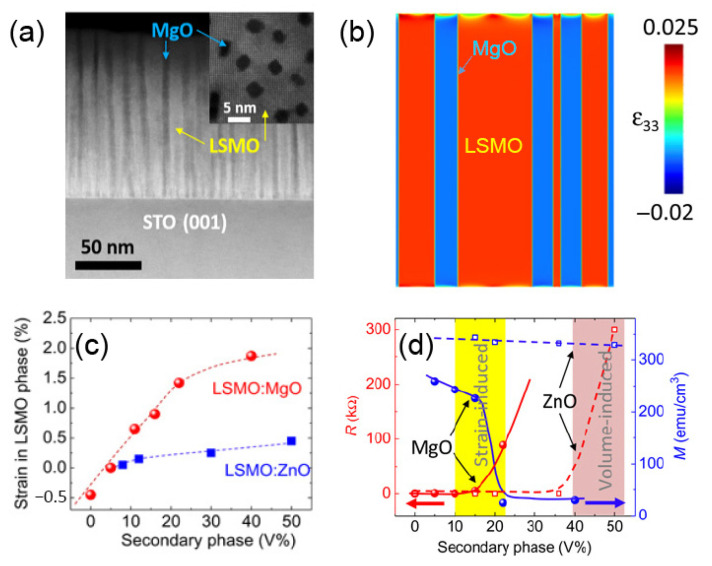
(**a**) A cross-sectional STEM image of an LSMO:MgO nanocomposite with phase-separated LSMO (white) and MgO (dark) phases. Inset: A top-view STEM image of the LSMO:MgO film with MgO nanopillars in the LSMO matrix. The MgO volume is 22%. (**b**) The strain distribution in the LSMO matrix and MgO nanopillars (MgO volume: 41%) calculated by phase-field simulation. (**c**) The second-phase volume-dependent vertical strain in the LSMO matrix. The red (dot) data represent the LSMO:MgO system, and the blue (square) data set represent the LSMO:ZnO system. (**d**) The second-phase volume-dependent resistance at 100 K (red dataset) and saturated magnetization (blue dataset) at 20 K for the LSMO:MgO (solid curve) and LSMO:ZnO (dashed curves) nanocomposite films. Reprinted with permission from ref. [32]. Copyright 2016, AAAS.

Chen et al. explored the vertical strain engineering of the La_0.7_Sr_0.3_MnO_3_ system via incorporating nanopillars of ZnO and MgO in the nanocomposites [32]. The strain in the La_0.7_Sr_0.3_MnO_3_:MgO nanocomposites was much larger than that in the La_0.7_Sr_0.3_MnO_3_:ZnO with the same volume of the second phase due to the larger lattice mismatch and elastic modulus mismatch. Figure 13 shows the comparative studies of the nanocomposites with different second-phase materials and volume ratios. The rise in resistivity for the La_0.7_Sr_0.3_MnO_3_:ZnO at ~50% of second-phase (ZnO) volume can be explained by a second-phase-induced volume effect. On the other hand, the sharp drop of over 10 times in resistivity of La_0.7_Sr_0.3_MnO_3_:MgO happens at only 20% second-phase (MgO) volume which is quite below the volume-induced percolation threshold. The experimental results, therefore, could predominantly be explained by the vertical strain effects. Vertical strain effects could also be the cause of the large drop in the metal-to-insulator transition temperature from 350 K of the bulk value of La_0.7_Sr_0.3_MnO_3_ to 150 K in the La_0.7_Sr_0.3_MnO_3_:MgO nanocomposite. 

It is also noted that the strain on the LSMO matrix could be tuned via the density and size of the MgO nanopillars [32]. This, consequently, will affect the magnetic properties of the LSMO. For example, the LSMO:MgO nanocomposite with 5% MgO inclusion showed an in-plane easy axis. With increasing the density of MgO nanopillars (15% MgO inclusion), the easy axis switches to the out-of-plane direction. This switching of the magnetic anisotropy is correlated by the strain on the LSMO matrix exerted by the MgO nanopillars.

## 6. Future Prospects

As discussed above, perovskite metal oxides have been considered as an extremely important class of materials for technological applications due to their versatile and tunable physical properties. At the same time, the unique interplay between the charge, spin, orbital and lattice degrees of freedom across similar energy scales also makes the physical properties of such materials very sensitive to both microstructural and compositional variations. This is understandable considering that the distortions of crystal structure resulting from the change in chemistry and/or the eight corner-shared [BO_6_] octahedra will lead to the change in the electronic structure of the materials. Over the years, tremendous progress in controlled synthesis, advanced characterization at different length and time scales, and sophisticated modeling/simulation have helped us to fundamentally understand the processing–structure–property–performance relationship of perovskite metal oxides. Even so, more effort is much needed to further explore and design perovskites with desired properties, given their rich compositional diversity where the properties are closely tied to the crystal distortion such as the lattice strain. Below are some interesting and yet challenging research activities for the development of perovskite materials with enhanced, desired, and/or emergent properties.

(1) An in-depth and quantitative understanding of the role of interface and defect on the properties is critical for the rational design of novel perovskite materials. This is especially important where superlattice and/or vertically aligned nanocomposite are the building blocks [11];
(2) Substitution of ions via chemical pressure is one of the common approaches to strain the crystal structure. Much improved properteis could be achieved by forming multicomponent or high entropy perovskite oxides [119];
(3) Epitaxial strain resulting from the lattice mismatch between the substrate and the film has been widely investigated for a range of perovskite metal oxide films. The exploration of dynamic strain by laminating freestanding perovskite metal oxide films or membranes onto a stretchable substrate could provide possibilities for strain-tunable devices [8];
(4) Quantitative analysis of oxygen vacancies on the structural and physical properties of perovskite metal oxides is much needed. This is especially important for perovskites that are composed of cations with multiple valance states at the B site [120];
(5) The formation of nanocomposite represents an attractive strategy to achieve tunable properties. More effort is needed to address the challenges related to the controlled synthesis to achieve long-range ordered structures [97];
(6) While the most commonly studied perovskites are complex metal oxides, other perovskite materials, namely the halide perovskite [121], chalcogenide perovskite [122], and nitride perovskite [123], have attracted much attention lately. Halide perovskites, in particular, have attracted quite a lot of attention due to their potential applications for optoelectronic devices such as solar cells. However, the strain analysis and engineering in these thin films and devices have significantly different considerations than that of metal oxide perovskites, due to the soft ionic nature of the crystal lattice, generally non-epitaxial, and the complex interfaces of different charge transport layers [124,125,126,127,128]. While many studies has been undertaken and tremendous progress has been made in the synthesis and application of such materials, there are still many open questions related to the roles of strain, defect, interface, microstructure, and heterogeneity at different length scales on the physical properties of the materials. The recent review article published by Liu et al. summarizes the progress and challenges on this front admirably [129].

## 7. Conclusions

Perovskite metal oxides exhibit a multitude of important physical properties, such as metal-to-insulator transitions, superconductivity, ferroelectric, ferromagnetic, and magnetoresistive properties. As these properties are intimately linked with the structure of the perovskite, factors such as octahedral rotations, tilts and distortions, and chemical substitution with different sized cations can be also used to exert a chemical pressure to tailor these properties. Epitaxial strain is another lever that can be used to tune these properties in thin films by choosing an appropriate substrate with an appropriate lattice mismatch. Multilayers and/or superlattices take the advantage of layer thickness and multiple lateral interfaces to control the strain state of the assembly. Vertically aligned nanocomposites are an exciting way to construct strained thick films that is not possible in lateral heterostructures. Importantly, novel or improved functionalities, often not accessible from the individual constituents, could be produced through synergistic coupling interactions of known materials and lattice strain along the vertical directions. In the vertically aligned nanocomposite, it is possible to select one constituent as the active phase (typically perovskite metal oxide) with targeted functionality, and another as the passive phase (usually binary metal oxide), where the passive constituent is used to improve and/or tune the functionalities of the active phase. Such a strategy makes it possible to choose a much broader range of materials with targeted electrical, magnetic, optical, thermal, and/or mechanical properties for specific applications. 

## Figures and Tables

**Table 1:** Summary of vertical lattice strain reported in different material systems with direct lattice-matching between the constituents *A* and *B*. For the direct lattice-matching of strained lattices, m lattices of phase *A* match with *K* lattices of phase *B* (m and k are positive integer numbers). The data was taken with permission from [32]. Copyright 2016, American Association for the Advancement of Science.

System	a_bulk_ [Å]	*K*	|f| [%]	|ε_zz_| [%]	Reference
LSMO:MgO	3.87:4.21	1	8.41	2.0	[32]
LCMO:MgO	3.86:4.21	1	8.67	2.1	[105]
BFO:CFO	3.96:8.39	2	5.76	1.0	[106]
BTO:CFO	4.04:8.39	2	3.76	1.6	[107]
BFO:LSMO	3.96:3.87	1	2.29	1.3	[108]
BZO:YBCO	11.679:4.193	3	7.70	1.0	[109]

**Table 2:** Summary of vertical lattice strain reported in different materials systems with domain-matching between the constituents *A* and *B*. The data was taken with permission from [32]. Copyright 2016, American Association for the Advancement of Science.

System	a_bulk_ [Å]	m	m:m + 1	|f| [%]	|ε_zz_| [%]	Reference
LSMO (001):ZnO (110)	3.87:3.24	5.14	5:6	0.46	≈0.5, 1.0	[110,111]
LSMO (111):ZnO (0001)	6.70:5.21	3.50	3:4 + 4:5	8.5 × 10−3	0	[112,113]
CeO_2_:LSMO *	5.41:3.87	2.51	2:3 + 3:4	0.13	<0.1	[114]
SrZrO_3_:Gd_2_O_3_ **	4.09:2.70	1.94	2:3	0.98	0.9	[115]
BTO:Sm_2_O_3_	4.04:2.73	2.08	2:3	1.17	2.35	[116]
BFO:Sm_2_O_3_	3.96:2.73	2.22	2:3 + 2:3 + 3:4	1.71	≈1.4	[110]
STO:Sm_2_O_3_	3.905:2.73	2.32	2:3 + 2:3 + 3:4	0.31	–	[117]

* The calculated m value is 2.51 for CeO_2_:LSMO; therefore, m can be valued as 2 or 3. Therefore, both 2:3 and 3:4 matchings exist and align alternatively (50%:50%). In the STO:Sm_2_O_3_ system, m is 2.32. m can be either 2 or 3. Both 2:3 and 3:4 matchings exist with a frequency of 66% 2:3 and 34% 3:4. In the SrZrO_3_:Gd_2_O_3_ system, the calculated m is 1.94; therefore, the m is set to 2 for domain-matching. ** Bulk lattice constant of Gd_2_O_3_ is 10.80 Å. The plane spacing for Gd_2_O_3_ (004) is 10.80 Å/4 = 2.7 Å.

## Data Availability

No new data were created or analyzed in this study. Data sharing is not applicable to this article.
